# Macitentan treatment retards the progression of established pulmonary arterial hypertension in an animal model^☆^

**DOI:** 10.1016/j.ijcard.2014.09.005

**Published:** 2014-12-15

**Authors:** I.P. Temple, O. Monfredi, G. Quigley, H. Schneider, M. Zi, E.J. Cartwright, M.R. Boyett, V.S. Mahadevan, G. Hart

**Affiliations:** a Institute of Cardiovascular Sciences, University of Manchester, UK; b Central Manchester University Hospitals NHS Trust, UK

**Keywords:** Pulmonary arterial hypertension, Pulmonary hypertension, Macitentan, Echocardiogram, ECG, Monocrotaline

## Abstract

**Background:**

Macitentan is a new endothelin receptor antagonist that is used to treat pulmonary arterial hypertension in humans. Treatment of established pulmonary hypertension with macitentan was studied using the monocrotaline model of pulmonary hypertension.

**Methods:**

Three groups of rats were created (n = 12): control (CON: macitentan only), monocrotaline (MCT: monocrotaline only) and macitentan (MACI: macitentan and monocrotaline). Monocrotaline (60 mg/kg) was injected in the MCT and MACI groups on day 0; volume matched saline was injected in the CON groups. Macitentan therapy (30 mg/kg/day) was commenced on day 11 in the CON and MACI groups. Serial echocardiography and ECGs were performed. The rats were sacrificed if they showed clinical deterioration.

**Results:**

The MCT and MACI rats showed signs of pulmonary hypertension by day 7 (maximum pulmonary velocity, CON 1.15 ± 0.15 m/s vs MCT 1.04 ± 0.10 m/s vs MACI 0.99 ± 0.18 m/s; p < 0.05). Both the MCT and MACI groups developed pulmonary hypertension, but this was less severe in the MACI group (day 21 pulmonary artery acceleration time, MCT 17.55 ± 1.56 ms vs MACI 22.55 ± 1.00 ms; pulmonary artery deceleration, MCT 34.72 ± 3.72 m/s^2^ vs MACI 17.30 ± 1.89 m/s^2^; p < 0.05). Right ventricular hypertrophy and QT interval increases were more pronounced in MCT than MACI (right ventricle wall thickness, MCT 0.13 ± 0.1 cm vs MACI 0.10 ± 0.1 cm; QT interval, MCT 85 ± 13 ms vs MACI 71 ± 14 ms; p < 0.05). Survival benefit was not seen in the MACI group (p = 0.50).

**Conclusions:**

Macitentan treatment improves haemodynamic parameters in established pulmonary hypertension. Further research is required to see if earlier introduction of macitentan has greater effects.

## Highlights

• Monocrotaline administration caused pulmonary hypertension in rats.
• Macitentan treatment delayed the progression of established pulmonary hypertension.
• Macitentan treatment reduced right ventricular hypertrophy.
• Macitentan treatment reduced QT interval prolongation.
• Macitentan treatment caused no adverse events in the control group.

## 1 Introduction

Pulmonary arterial hypertension (PAH) is a disease characterised by raised pulmonary vascular resistance. It has a poor prognosis typically resulting in progressive right ventricular failure and death. Treatment in PAH has advanced rapidly over the past decade with the use of Ca^2 +^ channel blockers, prostanoids, endothelin receptor antagonists (ERAs) and phosphodiesterase-5 inhibitors [1]. PAH often has an insidious onset, which means that diagnosis and treatment are usually not begun until the disease is advanced. Recent studies have looked at patients with less severe disease (World Health Organisation (WHO) class II), and have shown that early initiation of therapy can delay the progression of the disease [2].

Endothelin is a 21-amino acid peptide which is produced mainly by the vascular system and acts in a paracrine manner to regulate vasoconstriction, cell proliferation, cell migration and fibrosis [3]. Activation of the endothelin system plays a central role in the pathogenesis of PAH [3]. ERAs are widely used in clinical practice for patients with WHO class II to IV symptoms, either as monotherapy or in combination with other agents [1], [4], [5]. They have beneficial effects on haemodynamic parameters, objective measurements of exercise capacity and subjective symptom scores [1], [4], [5]. Data regarding ERAs and mortality are limited, although registry data suggests a survival benefit with the ERA bosentan [1], [4], [5]. Macitentan is a new ERA which has been shown in animal studies to have improved tissue penetration, longer receptor binding and affinity for both the endothelin A and B receptors compared with the older ERA bosentan [6]. The use of macitentan to treat PAH has been investigated in a phase III clinical trial enrolling WHO class II to IV patients showing a significant improvement in exercise capacity and haemodynamic parameters at 6 months and a significant reduction in morbidity over a followup period of up to 36 months [7].

Monocrotaline is a pyrrolizidine alkaloid, extracted from the plant *Crotalaria spectabilis*. A single injection has been shown to generate severe pulmonary hypertension in several species and has been widely used as an animal model of pulmonary hypertension in the rat [8], [9]. The effects of monocrotaline on pulmonary arterial pressures, pulmonary vascular resistance and right ventricular hypertrophy have been studied using invasive methods with direct pressure methods and non-invasive methods including echocardiography (echo) and magnetic resonance imaging (MRI) [10], [11]. These studies have demonstrated a characteristic change in the pulmonary velocity profile from the typical rounded shape to a ‘spike and dome’ morphology [10], [11]. The echo parameter ‘pulmonary artery deceleration’ (PAD) is correlated to pulmonary arterial pressure measured invasively and the echo parameter ‘pulmonary artery acceleration time’ (PAAT) is inversely correlated to both pulmonary pressure and pulmonary vascular resistance measured invasively [10], [11].

Experiments using the monocrotaline model have given positive results from drug therapy including ERAs, sildenafil, statins and beta blockers [6], [12], [13], [14], [15]. The experimental design of these studies has varied such that some studies have started therapy on the same day as the monocrotaline injection, i.e. a ‘prevention’ strategy, whereas others have waited until there is evidence of the animals displaying pulmonary hypertension before commencing therapy, i.e. a ‘treatment’ strategy. In cases where ‘prevention’ and ‘treatment’ have been compared there has been a greater effect with ‘prevention’ than with ‘treatment’ [13], [14], [16]. These findings raise questions about the extent to which ‘prevention’ studies are applicable to clinical practice, particularly given that the dramatic successes seen in ‘prevention’ studies have not been borne out in clinical practice.

Animal studies with macitentan administration, given as a ‘prevention’ strategy, have shown a significant mortality benefit. In order to more closely to reflect current clinical practice, we have investigated the safety and efficacy of macitentan administration *after* the development of pulmonary hypertension in the monocrotaline model.

## 2 Methods

All procedures were carried out in accordance with the UK Animals Scientific Procedures Act (1986). Invasive pulmonary pressure monitoring in monocrotaline injected rats has demonstrated that pulmonary pressures are significantly raised by day 10 and increase progressively, leading to RV failure and death [11], [17]. In the light of such previous studies we elected to initiate therapy at day 11, in order to mirror the clinical situation with respect to initiation of treatment. Male Wistar Harlan rats (n = 36; weight 200 g; Charles River, UK) were arbitrarily assigned to three equal groups (n = 12). All animals received pulverised chow only from day 0 to day 11. The control group (CON) received saline injection (3 ml/kg) by intraperitoneal injection on day 0 and macitentan (Actelion Pharmaceuticals Ltd, Allschwil, Switzerland) 30 mg/kg/day admix to pulverised chow from day 11 to the day of termination. The monocrotaline only group (MCT) received monocrotaline 60 mg/kg by intraperitoneal injection on day 0 and pulverised chow only from day 11 to the day of termination. The macitentan treated group (MACI) received monocrotaline injection 60 mg/kg by intraperitoneal injection on day 0 and macitentan 30 mg/kg/day admix to pulverised chow day 11 to the day of termination [6]. Monocrotaline (Sigma-Aldrich Ltd, UK) was dissolved in 1 M hydrochloric acid, then made up to a concentration of 20 mg/ml with 0.9% saline, the pH corrected to 7.4 using 4 M NaOH.

ECG and echo recording was carried out under general anaesthesia with 2% isoflurane. Electrodes were inserted subcutaneously with the negative electrode in the right forepaw, the positive electrode in the left forepaw and the ground electrode in the right hindpaw. The electrodes were connected to a Bioamp and Powerlab analogue to digital converter (AD instruments, New Zealand). Signals were recorded using Labchart (AD Instruments, New Zealand) and analysed offline. All intervals were measured from the average of 100 beats using Chart software. ECG was recorded on day 0 immediately prior to injection, and on day 7, day 14 and day 21. QTc was calculated using Bazett's formula.

**Fig. 1:**
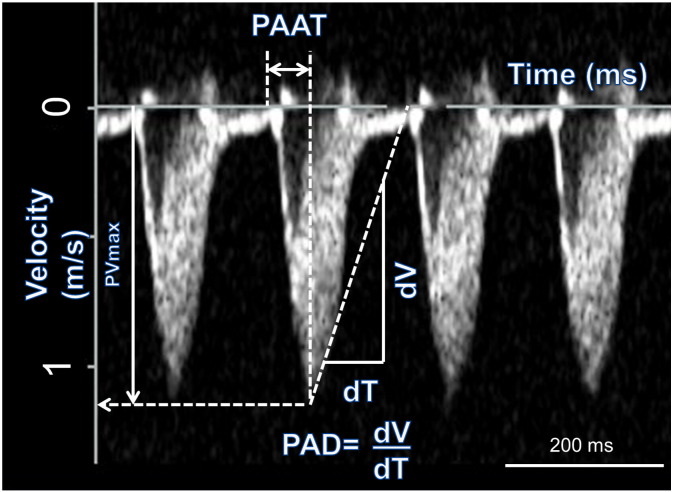
Pulsed wave Doppler recording through the pulmonary artery and measurement of PVmax, PAAT and PAD. The x axis measures time and the y axis measures velocity. PAAT is the time from the beginning of flow to the peak velocity, measured from the x axis. PVmax is the maximum velocity measured from the y-axis. PAD is the gradient of the initial deceleration of the pulmonary velocity profile.

Echo images were acquired on an ACUSON Sequoia™ (Acuson Universal Diagnostics Solution, USA) with a 15 MHz 15L8 transducer. All images were stored on optical media disks for subsequent offline analysis. M-mode recordings were taken in the parasternal short axis view allowing recording of left ventricle (LV) anterior and posterior wall thickness and the internal diameter of the LV in both systole and diastole. Right ventricle (RV) wall thickness was measured from M-mode recordings in the parasternal long axis view. Continuous wave Doppler recordings through the pulmonary artery were used to assess the pulmonary velocity profile. The maximum pulmonary velocity (PVmax), time from the onset of pulmonary outflow to maximal flow (pulmonary artery acceleration time, PAAT) and the rate of deceleration of pulmonary flow (pulmonary artery deceleration time, PAD) were measured (Fig. 1). Echo was recorded on day 0 immediately prior to injection, on day 7, day 14, and day 21.

### 2.1 Symptomatic endpoints

The animals were weighed and their clinical condition was assessed twice weekly in the first 18 days, and daily thereafter. Animals were sacrificed on the day that the following pre-specified endpoints were met, namely evidence of clinical deterioration with reduced movement, increased respiratory rate, piloerection and weight loss of > 10 g over 2 days. Animals that did not meet these criteria were electively sacrificed on day 28. The animals were sacrificed by stunning and cervical dislocation; the heart and lungs were excised and weighed.

### 2.2 Statistical methods

The distribution of the data was analysed using the Shapiro–Wilk test. The data were found to be normally distributed and therefore analysis of the differences between body weight, heart weight and lung weight was performed using Student's *t*-test. Comparisons of the echo and ECG parameters at day 0, day 7, day 14 and day 21 were made using a two-way repeated measures ANOVA with the two factors being time and treatment group; time was the repeated measure. Comparisons were made between the three treatment groups at each timepoint using the Tukey test to correct for multiple comparisons. Survival analysis was performed using the log-rank (Mantel–Cox) test.

## 3 Results

Table 1 shows that both the MCT group and the MACI group had increased heart weight and decreased body weight compared with the CON group. The differences between the MCT group and MACI group were not significant. There was no difference in lung weight between the MACI treated group and the CON group. Although the MCT group did show an increase in lung weight compared with the CON group, there was no significant difference when the MCT group and MACI group were compared directly.

**Table 1:** Body weights on day 21 and heart and lung weights on termination of the CON, MCT and MACI groups.

	CON (n = 12)	MCT (n = 11–12)	MACI (n = 12)	p-Value for MCT V MACI
Body weight at day 21 (g)	359 ± 11	331 ± 6⁎	328 ± 6⁎	0.70
Heart weight (g)	1.24 ± 0.12	1.541 ± 0.21⁎⁎	1.46 ± 0.14⁎⁎	0.28
Lung weight (g)	2.36 ± 1.08	3.206 ± 0.71⁎	2.85 ± 0.35	0.14

⁎ p < 0.05 MCT or MACI vs CON.

⁎⁎ p < 0.005 MCT or MACI vs CON.

**Fig. 2:**
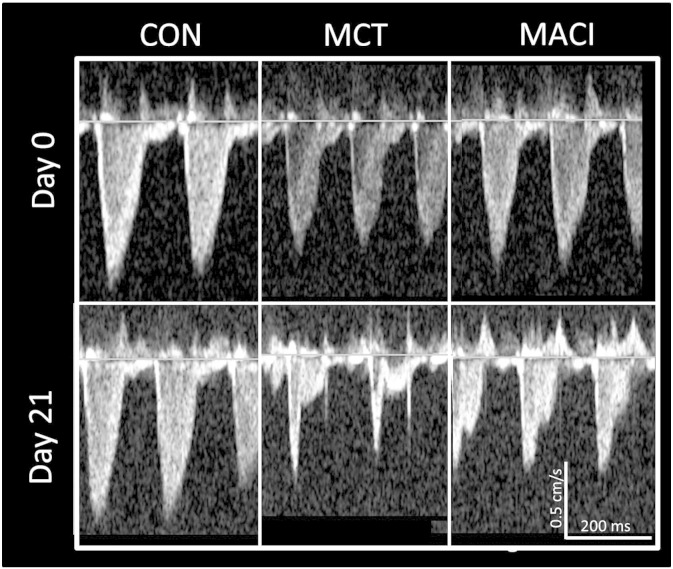
Echo images showing the development of pulmonary hypertension assessed by the pulmonary velocity profile. The profile has a typical ‘rounded’ shape prior to injection. At day 21 no change is seen in the CON animal but the MCT animal shows a change to a typical ‘spike and dome’ morphology with a reduced PAAT and increased PAD. The MACI animal has an intermediate profile between the two groups.

**Fig. 3:**
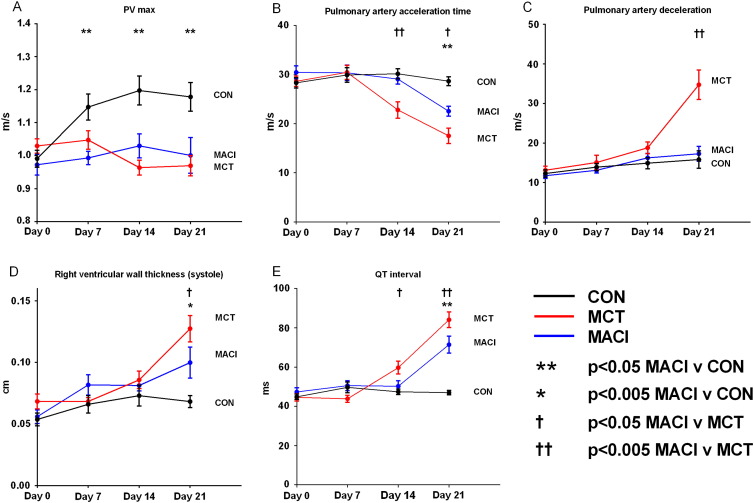
Development of pulmonary hypertension. Mean and SEM plotted at each time point. The MACI group was compared with the MCT group and the CON group by a *t*-test. Pulmonary hypertension developed in the MACI group by day 7 (i.e. before the initiation of macitentan) with a reduced PVmax. There is a significant improvement in the MACI group compared to the MCT group at day 14 and day 21 with a reduced PAD, right ventricular wall thickness in systole and QT interval and an increased pulmonary artery acceleration time.

Fig. 2 shows that both the MCT and MACI group developed echo evidence of pulmonary hypertension with a change from a ‘rounded’ pulmonary velocity profile to a ‘spike and dome’ morphology. The timings of these changes are summarised in Fig. 3. The earliest changes in echo parameters were seen at day 7 with a reduction in PVmax of 9% in the MCT group and 13% in the MACI group compared with the CON group (Fig. 3A). No other parameters were significantly altered by day 7. The reduction of PVmax in both MCT and MACI groups when compared with the CON group suggests that pulmonary hypertension had begun to develop by day 7.

At day 14 there was evidence of pulmonary hypertension in both the MCT and MACI groups, with reductions in PVmax of 20% in the MCT group and 14% in the MACI group compared to the CON group. The PAAT was reduced by 25% in the MCT group compared with the CON group and 22% compared with the MACI group. There was no significant change in PAAT in the MACI group compared with the CON group. No other significant changes were seen between the MACI and MCT groups (Table 2; Fig. 3).

**Table 2:** Echo measurements on day 14 for CON, MCT and MACI groups.

	CON (n = 11/12)	MCT (n = 11/12)	MACI (n = 11/12)	p-Value for MCT V MACI
PAAT ms	30.2 ± 1.0	22.8 ± 1.7⁎⁎	29.1 ± 0.9††	0.00
PAD m/s^2^	14.9 ± 1.4	18.8 ± 1.5	16.3 ± 1.2†	0.55
PVmax m/s	1.20 ± 0.04	0.96 ± 0.02⁎⁎	1.03 ± 0.03⁎⁎	0.39
RV internal diameter (diastole) (cm)	0.16 ± 0.03	0.17 ± 0.02	0.22 ± 0.02	0.34
RV internal diameter (systole) (cm)	0.07 ± 0.01	0.08 ± 0.02	0.10 ± 0.01	0.82
RV wall thickness (diastole) (cm)	0.05 ± 0.00	0.06 ± 0.01	0.06 ± 0.00	0.96
RV wall thickness (systole) (cm)	0.07 ± 0.01	0.09 ± 0.01	0.08 ± 0.00	0.90
LV internal diameter (diastole) (cm)	0.75 ± 0.01	0.70 ± 0.02	0.70 ± 0.01⁎	0.99
LV internal diameter (systole) (cm)	0.34 ± 0.01	0.32 ± 0.02	0.30 ± 0.02	0.87
LV anterior wall thickness (diastole) (cm)	0.17 ± 0.00	0.18 ± 0.01	0.18 ± 0.01	0.99
LV anterior wall thickness (systole) (cm)	0.29 ± 0.01	0.31 ± 0.01	0.34 ± 0.02	0.99

Table showing echo parameters of pulmonary hypertension at day 14.

⁎ p < 0.05 MCT or MACI vs CON.

⁎⁎ p < 0.005 MCT or MACI vs CON.

† p < 0.05 MCT vs MACI.

†† p < 0.05 MCT vs MACI.

At day 21, both the MCT and MACI groups continued to show evidence of pulmonary hypertension with a reduction in PAAT of 39% in the MCT group and 22% in the MACI group compared with the CON group. The rise in RV pressure caused compression of the LV which resulted in a decrease in LV internal diameter in both diastole and systole in both the MCT and MACI groups (Table 3). Although the MACI group continued to show evidence of pulmonary hypertension, this was less severe than in the MCT group; the MACI group had a 29% greater PAAT and a 50% lower PAD than the MCT group. Further demonstration that pulmonary hypertension was less severe in the MACI group than the MCT group was the finding that the RV wall thickness in systole was 23% smaller in the MACI group (Table 3; Fig. 3B–D).

**Table 3:** Echo measurements for day 21 of CON, MCT and MACI groups.

	CON (n = 11/12)	MCT (n = 11)	MACI (n = 11/12)	p-Value for MCT V MACI
PAAT ms	28.7 ± 0.9	17.5 ± 1.6⁎⁎	22.5 ± 1.0⁎⁎, †	0.02
PAD m/s^2^	15.8 ± 2.2	34.7 ± 3.7⁎⁎	17.3 ± 1.9††	0.00
PVmax m/s	1.18 ± 0.04	0.97 ± 0.03⁎⁎	1.00 ± 0.05⁎⁎	0.80
RV internal diameter (diastole) (cm)	0.16 ± 0.02	0.27 ± 0.03⁎⁎	0.21 ± 0.02	0.13
RV internal diameter (systole) (cm)	0.08 ± 0.02	0.14 ± 0.04⁎	0.12 ± 0.03	0.66
RV wall thickness (diastole) (cm)	0.05 ± 0.00	0.09 ± 0.01⁎⁎	0.07 ± 0.01	0.12
RV wall thickness (systole) (cm)	0.07 ± 0.01	0.13 ± 0.01⁎⁎	0.10 ± 0.01⁎, †	0.02
LV internal diameter (diastole) (cm)	0.78 ± 0.01	0.66 ± 0.04⁎⁎	0.68 ± 0.02⁎⁎	0.64
LV internal diameter (systole) (cm)	0.39 ± 0.02	0.26 ± 0.03⁎⁎	0.27 ± 0.02⁎⁎	0.96
LV anterior wall thickness (diastole) (cm)	0.17 ± 0.01	0.21 ± 0.02	0.21 ± 0.01	0.94
LV anterior wall thickness (systole) (cm)	0.29 ± 0.01	0.37 ± 0.03⁎⁎	0.34 ± 0.02	0.18

Table showing echo parameters of pulmonary hypertension at day 21.

⁎ p < 0.05 MCT or MACI vs CON.

⁎⁎ p < 0.005 MCT or MACI vs CON.

† p < 0.05 MCT vs MACI.

†† p < 0.05 MCT vs MACI.

In vivo ECG recordings showed no significant changes in any parameters at day 7. At day 14, the QT interval was prolonged in the MCT group by 26% compared with the CON group and by 18% compared with the MACI group. The QT_C_ interval was prolonged in the MCT group by 23% compared with the CON group. There difference in QT_C_ between the MACI group and both the CON group and MCT group were not significant. At day 21, QT interval was increased by 79% in the MCT group and by 52% in the MACI group compared with the CON group. QT interval was shorter by 15% in the MACI group than in the MCT group. Similarly at day 21, QT_C_ interval was increased by 75% in the MCT group and by 48% in the MACI group compared with the CON group. QT_C_ interval was shorter by 15% in the MACI group than in the MCT group. There were no changes seen in any of the other ECG parameters (Table 4).

**Table 4:** ECG measurements on day 21 or CON, MCT and MACI groups.

	CON (n = 12)	MCT (n = 11)	MACI (n = 12)	p-Value for MCT V MACI
Heart rate (bpm)	393.50 ± 7.77	372.10 ± 8.10	380.50 ± 12.15	0.79
PR interval (ms)	48.54 ± 3.29	48.21 ± 4.705	47.87 ± 4.68†	0.92
QRS duration (ms)	15.29 ± 2.58	14.07 ± 1.40	14.33 ± 1.17	0.52
QT interval (ms)	47.00 ± 3.99	84.56 ± 12.55⁎⁎	70.69 ± 13.95⁎⁎,††	0.00
QT_C_ interval (ms)	120 ± 3.14	210.3 ± 9.34⁎⁎	178.1 ± 10.81⁎⁎,††	0.00

Table showing ECG measurements at day 14.

⁎⁎ p < 0.005 MCT or MACI vs CON.

† p < 0.05 MCT vs MACI.

†† p < MCT vs MACI.

**Fig. 4:**
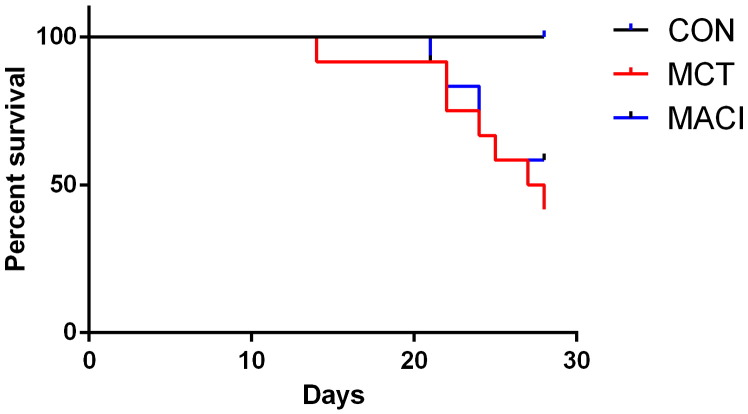
Kaplan–Meir curves showing the freedom from symptomatic endpoints. The animals were sacrificed on the day they met their symptomatic endpoints. There is a significant difference between the CON and MACI treated groups (p = 0.01) but no difference between the MACI treated and MCT groups (p = 0.50).

Freedom from symptomatic endpoints at day 28 was 100% in the CON group, 42% in the MCT group and 58% in the MACI group. There was a significantly worse survival in both the MCT and MACI groups when compared with the CON group (MCT p = 0.002, MACI p = 0.014). The difference in freedom from symptomatic endpoints between the MCT and MACI groups was not statistically significant (p = 0.50) (Fig. 4).

## 4 Discussion

This study shows for the first time that treatment with the ERA macitentan retards and delays the progression of established pulmonary hypertension in the rat monocrotaline model. The pulmonary pressures in the three groups have been estimated using the echo parameters PAD and PAAT. As described in the Introduction section both an increase in PAD and a decrease in PAAT correlate with an increase in pulmonary artery pressure [10], [11]. The earliest signs of pulmonary hypertension developing are seen by day 7 with a reduction of PVmax seen in both the MCT and MACI groups. Macitentan treatment was initiated at day 11, and the data show significant slowing of progression of the echo and ECG parameters of pulmonary hypertension by as early as day 14 with a greater PAAT in the MACI group than the MCT group (Fig. 3B). The MACI group showed less severe parameters of pulmonary hypertension than the MCT group at both day 14 and day 21 (Fig. 3). Despite the early beneficial effects of macitentan on pulmonary pressures in the MACI group compared with the MCT group the evidence suggests that the pulmonary pressures are increased in the MACI group compared with the CON group by day 21 with a significant reduction in PAAT demonstrated in the MACI group. Delaying the progression of pulmonary hypertension resulted in reduced structural and electrical remodelling with improvements in right ventricular wall thickness and QT interval at day 21 (Table 4; Fig. 3E) in the MACI group compared with the MCT group. These findings suggest that treatment with macitentan is able to improve haemodynamic parameters in animals with established pulmonary hypertension in the earlier stages of the disease. However, the underlying pathogenesis of the monocrotaline model continues to progress, and pulmonary hypertension continues to develop despite macitentan treatment (Fig. 3, Fig. 4).

In spite of increasing interest in treating patients with PAH earlier in their disease course, advanced therapies for PAH are considered only in patients who have proven raised pulmonary vascular resistance and symptoms [1], [2]. In our study, the earliest indication of pulmonary hypertension was seen at day 7 with a reduced PVmax, and there was clear evidence of pulmonary hypertension in the untreated monocrotaline group at day 14. This suggests that our experimental protocol is comparable to the clinical situation.

Previous studies using other compounds to treat pulmonary hypertension in the monocrotaline model including statins, endothelin blockade, rapamycin and vasoactive intestinal peptide have shown that the timing of initiation of therapy is crucial [13], [14], [16], [18], [19]. In the case of anti-proliferative therapy such as rapamycin early therapy has been shown to prevent the development of pulmonary hypertension, but to be ineffective if given after pulmonary hypertension has developed [14]. Therapy with ERAs initiated at the time of monocrotaline injection has a dramatic effect with a survival benefit, although it has been suggested that early therapy may delay rather than stop the development of pulmonary hypertension [16], [18], [19]. This can be compared with initiation of bosentan after pulmonary hypertension has developed, which has demonstrated more modest improvements in haemodynamic parameters and equivocal survival benefit [18].

The increase in heart weight seen in both the MCT and MACI groups is consistent with previous studies in the monocrotaline model that have demonstrated RV hypertrophy and an increased RV weight [12], [16]. Similarly the increase in lung weight seen in the MCT group has previously been demonstrated in the lungs in association with an inflammatory infiltrate [16]. There was no statistically significant difference in the lung weight in the MACI group compared with the CON group suggesting a relative decrease in inflammation within the lungs of the MACI group compared with the MCT group, but direct comparison did not show a difference between the MCT and MACI groups. The reduction in body weight in both the MCT and MACI groups compared with the CON group is again in keeping with previous studies and was used as a marker of clinical deterioration in the animal [12].

The mechanism by which monocrotaline treatment leads to pulmonary hypertension is still debated [3]. The initial insult is thought to occur in the pulmonary endothelial cells provoking an inflammatory response [9]. Progressive smooth muscle medial hypertrophy takes place from day ~ 4, and increases progressively until day 15 [9], [20]. Circulating levels of endothelin are raised in the monocrotaline model and these raised levels are not affected by ERAs, even if these drugs are given at the same time as the monocrotaline injection [19]. The beneficial effects of ERAs are thought to be attributable to a combination of improved endothelial function, vasodilating properties and a reduction of smooth muscle hypertrophy within the media of the pulmonary vessels [3], [18], [19], [21], [22]. Given the importance of inflammation and medial hypertrophy in the early stages of the monocrotaline model it is tempting to suggest that macitentan treatment may limit the pro-inflammatory and proliferative effects of endothelin if it is given early in the model development, before pulmonary hypertension has developed. The relatively rapid improvement in haemodynamic parameters seen in our study suggests that the vasodilating effects of endothelin blockade may be more relevant in established pulmonary hypertension as was present in our study.

QT_C_ prolongation has been demonstrated in both human and animal studies of pulmonary hypertension [23], [24], [25], [26]. In patients with PAH the QT_C_ is raised and a QT_C_ of greater than 480 ms is an independent predictor of mortality [25]. In addition to this the QT_C_ is correlated to pulmonary pressures and right ventricular dilation and inversely correlated to right ventricular function [25]. Animal studies have shown that the prolongation of QT interval is due to a reduction in repolarizing K^+^ currents and a prolongation of the ventricular action potential and that this can provide a substrate for ventricular arrhythmias [23], [24], [26]. The relative improvement in QT and QT_C_ interval in the MACI group suggests that macitentan may act to reduce the risk of arrhythmias as well as improving haemodynamic parameters.

The CON group received macitentan treatment in order to assess the safety profile of macitentan. There were no adverse events seen in the CON group confirming the safety of macitentan. The echo and ECG findings in the CON group were comparable to previous studies and our own unpublished data in which control groups received saline injections and no treatment, and would be consistent with the notion that macitentan treatment has no major effects on animals without pulmonary hypertension [27].

The results from our study are in keeping with clinical trials of PAH, in which despite early functional improvements, clear mortality benefit has not been shown [1]. In clinical practice PAH has an insidious presentation and diagnosis is typically late. Therefore the relevance of animal studies in which treatment is commenced before the development of pulmonary hypertension to clinical practice is debatable. The results of our study suggest that treatment with macitentan may offer benefits in terms of haemodynamic parameters, right ventricular function and QT prolongation. The relative contribution of anti-inflammatory, anti-proliferative and vasodilating actions of ERAs to treatment of PAH at different timepoints in the disease process are yet to be determined.

### 4.1 Study limitations

The safety of macitentan was demonstrated by the lack of adverse events in the CON group. A more robust analysis of the effects of macitentan on animals without pulmonary hypertension would require the addition of a control group that received a saline injection and no macitentan. However, the main aim of the study was to compare the differences between the untreated pulmonary hypertension in the MCT group and the treated pulmonary hypertension in the MACI group. This study demonstrates that macitentan treatment delays the progression of MCT induced pulmonary hypertension compared to the untreated animals. Similar benefits have also been shown with other therapies including the ERA bosentan and the phosphodiesterase-5 inhibitor sildenafil [12], [13], [16], [18].

We elected to use echocardiography to monitor the progression of pulmonary hypertension on the basis that it allows serial non-invasive measures, which have previously been validated against invasive pressure measurements in the right ventricle. Direct measurement of right-sided pressures and pulmonary vascular resistance may allow greater sensitivity to detect the effects of macitentan treatment, but are impractical for serial measurements.

Macitentan was administered via food admix to animal cages housing four rats. The dose was adjusted to animal weight and food intake was monitored to ensure that the correct dose of macitentan was being consumed on average between the four rats. However, it was not possible to ensure that each individual rat received exactly the specified dose. When the animals deteriorated their food intake may have diminished. However, the earliest deterioration in the MACI group was seen on day 21, meaning that the animal would have received macitentan treatment for a minimum of 10 days.

It is interesting to note that despite the use of the same dose regime of monocrotaline to that of Iglarz et al. we have seen a considerably more severe phenotype in the MCT group than previously demonstrated. In our study, 28-day freedom from symptomatic endpoints was 42% compared with a survival at day 28 of approximately 90% in their experiments [6]. Although these outcomes are not directly comparable the severe phenotype seen in our experiments may be attributable to the relatively low body weight of the rats at the time of monocrotaline injection. Therefore the benefits of macitentan therapy may to some extent have been masked by the severity of the MCT model phenotype, due to more rapid progression of the underlying lung pathology together with a shorter treatment duration before symptomatic endpoints were reached.

In conclusion, this study shows that treatment with macitentan provides worthwhile haemodynamic benefits in established pulmonary hypertension in association with reduced progression of the disease process. Taken together with others' data our results reinforce the notion that greater benefit may occur when treatment is initiated earlier in the course of the disease.

## Conflict of interest

VSM has previously received funding for a research fellowship from Actelion Pharmaceuticals for a different project.
